# Testing telediagnostic obstetric ultrasound in Peru: a new horizon in expanding access to prenatal ultrasound

**DOI:** 10.1186/s12884-021-03720-w

**Published:** 2021-04-26

**Authors:** Marika Toscano, Thomas J. Marini, Kathryn Drennan, Timothy M. Baran, Jonah Kan, Brian Garra, Ann M. Dozier, Rafael L. Ortega, Rosemary A. Quinn, Yu T. Zhao, Miguel S. Egoavil, Lorena Tamayo, Claudia Carlotto, Benjamin Castaneda

**Affiliations:** 1 Department of Obstetrics & Gynecology, Division of Maternal/Fetal Medicine, University of Rochester Medical Center, 601 Elmwood Ave, Box 668, Rochester, NY 14642 USA; 2 Department of Imaging Sciences, University of Rochester Medical Center, 601 Elmwood Ave, Box 648, Rochester, NY 14642 USA; 3 University of Rochester School of Medicine and Dentistry, 601 Elmwood Ave, Box 607, Rochester, NY 14642 USA; 4 Medical Imaging Ministries of the Americas, 10810 Lake Minneola Shores, Clermont, FL 34711 USA; 5 Department of Public Health Sciences, University of Rochester Medical Center, 265 Crittenden Blvd., Rochester, NY 14642 USA; 6 Medical Innovation and Technology, Calle Los Libertadores 635, 15046 San Isidro, Peru; 7 Departament of Academic Engineering, Division of Electric Engineering, Pontificia Universidad Catolica del Peru, Av. Universitaria 1801, 15088 San Miguel, Peru

**Keywords:** Global health, Handheld ultrasound, Low- and middle-income countries, Low-resource setting, Perinatal morbidity and mortality, Point-of-care ultrasound, Portable ultrasound, rural medicine, Telemedicine, Volume sweep imaging

## Abstract

**Background:**

Ninety-four percent of all maternal deaths occur in low- and middle-income countries, and the majority are preventable. Access to quality Obstetric ultrasound can identify some complications leading to maternal and neonatal/perinatal mortality or morbidity and may allow timely referral to higher-resource centers. However, there are significant global inequalities in access to imaging and many challenges to deploying ultrasound to rural areas. In this study, we tested a novel, innovative Obstetric telediagnostic ultrasound system in which the imaging acquisitions are obtained by an operator without prior ultrasound experience using simple scan protocols based only on external body landmarks and uploaded using low-bandwidth internet for asynchronous remote interpretation by an off-site specialist.

**Methods:**

This is a single-center pilot study. A nurse and care technician underwent 8 h of training on the telediagnostic system. Subsequently, 126 patients (68 second trimester and 58 third trimester) were recruited at a health center in Lima, Peru and scanned by these ultrasound-naïve operators. The imaging acquisitions were uploaded by the telemedicine platform and interpreted remotely in the United States. Comparison of telediagnostic imaging was made to a concurrently performed standard of care ultrasound obtained and interpreted by an experienced attending radiologist. Cohen’s Kappa was used to test agreement between categorical variables. Intraclass correlation and Bland-Altman plots were used to test agreement between continuous variables.

**Results:**

Obstetric ultrasound telediagnosis showed excellent agreement with standard of care ultrasound allowing the identification of number of fetuses (100% agreement), fetal presentation (95.8% agreement, *κ* =0.78 (*p* < 0.0001)), placental location (85.6% agreement, *κ* =0.74 (*p* < 0.0001)), and assessment of normal/abnormal amniotic fluid volume (99.2% agreement) with sensitivity and specificity > 95% for all variables. Intraclass correlation was good or excellent for all fetal biometric measurements (0.81–0.95). The majority (88.5%) of second trimester ultrasound exam biometry measurements produced dating within 14 days of standard of care ultrasound.

**Conclusion:**

This Obstetric ultrasound telediagnostic system is a promising means to increase access to diagnostic Obstetric ultrasound in low-resource settings. The telediagnostic system demonstrated excellent agreement with standard of care ultrasound. Fetal biometric measurements were acceptable for use in the detection of gross discrepancies in fetal size requiring further follow up.

**Supplementary Information:**

The online version contains supplementary material available at 10.1186/s12884-021-03720-w.

## Background

Globally, more than 800 women die each day from complications of pregnancy or childbirth, and for each death, 20 women suffer serious morbidity [1]. The majority of these maternal deaths occur in low- and middle-income countries (LMICs) and are mostly preventable [2, 3]. Access to quality Obstetric ultrasound can identify some complications leading to maternal mortality or morbidity and may allow timely referral to higher-resource centers [4–9]. Unfortunately, Obstetric ultrasound remains unavailable in two-thirds of the world, particularly in regions with the highest maternal morbidity and mortality [10, 11]. Increased use of Obstetric teleultrasound in rural areas could contribute towards improving these outcomes, and several reports have appeared in the recent literature demonstrating this [6, 12–24].

**Fig. 1:**
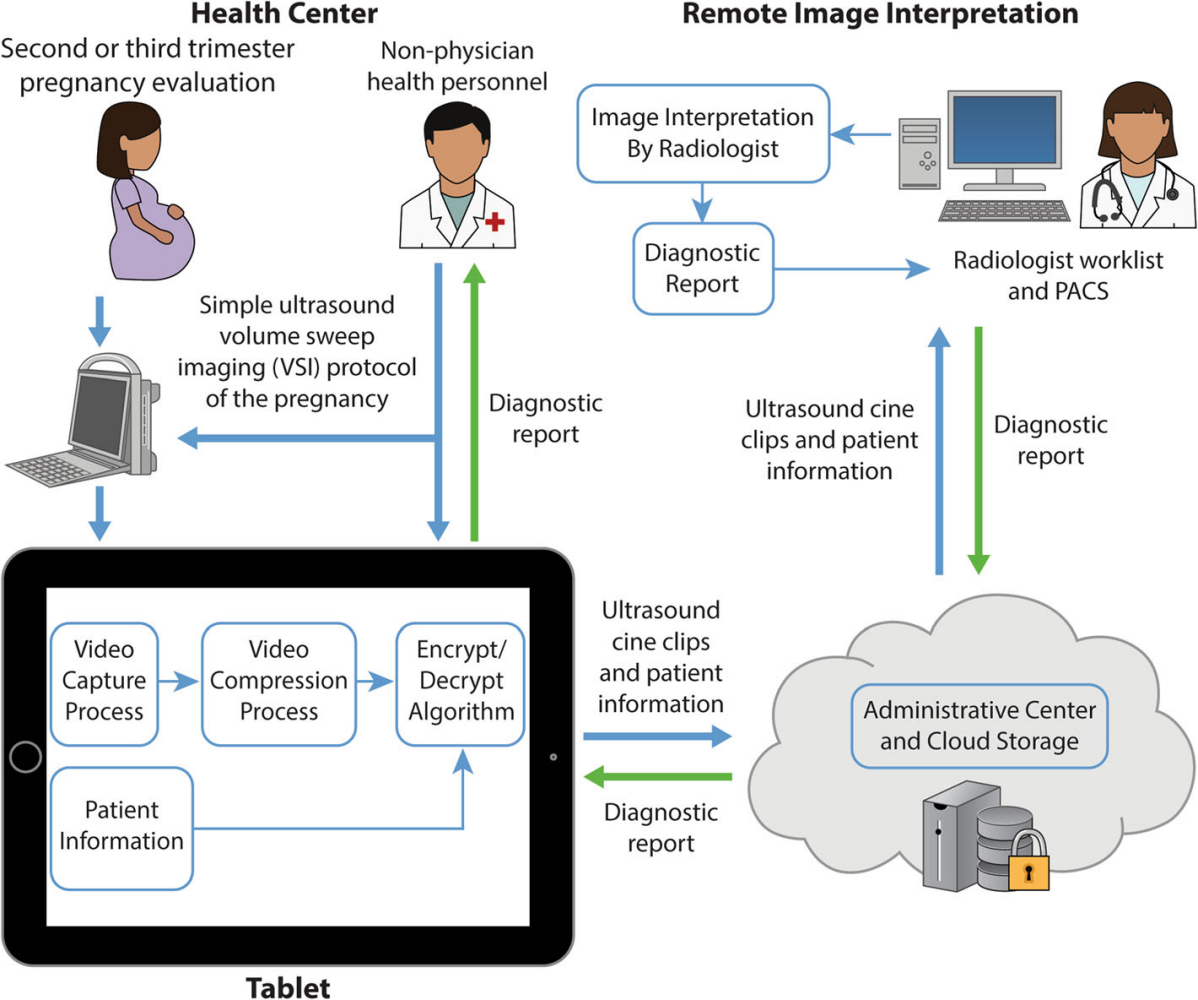
Overview of the Obstetric Telediagnostic System

Deploying ultrasound to rural areas is limited by many factors including lack of trained sonographers and sonologists, lack of appropriate equipment, and lack of infrastructure [8, 11, 13, 15, 25]. To overcome these challenges, we developed a novel telediagnostic ultrasound system which acquires images in the absence of a specialist (Fig. 1) [15]. Operators without prior ultrasound experience obtain cine clips of the target region using scan protocols that rely only on external body landmarks for proper transducer placement to obtain adequate imaging of anatomy and pathology. Acquisitions are sent using a telemedicine platform requiring low internet bandwidth for asynchronous remote interpretation.

A previous pilot study of this system for Obstetric teleultrasound in the rural rainforest of Peru demonstrated encouraging results but was limited by small sample size and lack of standard imaging for comparison [15]. Therefore, the goal of this study was to test the system in comparison to standard imaging in a larger sample of Peruvian women. The primary endpoint was to obtain sufficient data to test agreement in descriptive variables between the telediagnostic system (interpreted remotely) and standard of care (SOC) ultrasound (interpreted locally). Evaluation of image quality and calculation of intraclass correlation coefficients (ICC) for fetal biometry were also studied. We hypothesized that the system would yield diagnostic interpretations in agreement with SOC ultrasound and successfully identify critical features of the pregnancy.

## Methods

### Obstetric volume sweep imaging (VSI)

Obstetric volume sweep imaging (VSI) is a simple ultrasound examination technique in which the operator sweeps and arcs the transducer over the gravid abdomen to obtain cine clips containing a full volumetric acquisition of the target region. The ideal imaging speed is 1–2 cm/s, and an acquisition rate of 10–15 frames per second yields an image every 1–2 mm [15]. No specialized anatomical or ultrasound knowledge is necessary to perform the VSI protocol, therefore any individual may serve as an operator. The probe motions are simple, and ultrasound presets obviate the need for image adjustments. All image interpretation is done remotely by a specialist.

**Fig. 2:**
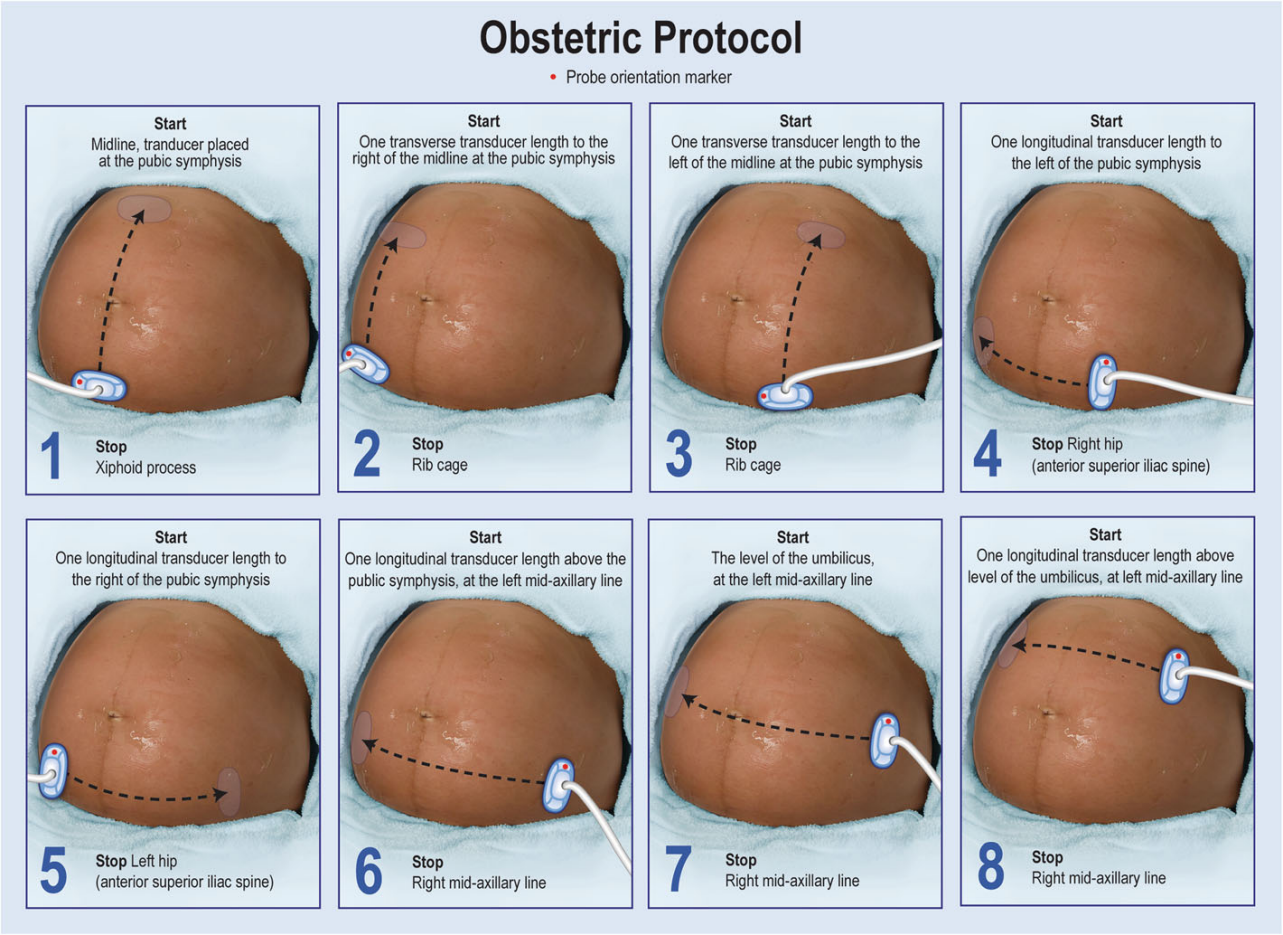
Illustration of the Obstetrics VSI Protocol. The Obstetrics protocol involves 8 sweeps each beginning and ending with an arc (fan) of the probe that has not been illustrated for simplicity. The arcing of the probe allows maximal visualization. Steps 1-3 involve transverse sweeps of the probe from the pelvis to the upper abdomen. Steps 4 and 5 are sagittal sweeps at the base of the pelvis. Steps 6-8 are sagittal sweeps on the upper gravid abdomen

The 8-step Obstetric VSI protocol (Fig. 2) was developed for simplicity, image redundancy, and optimal acoustic windows. Training an operator on the protocol can be accomplished effectively in a short time (in our experience, 8 h is sufficient to demonstrate mastery of the scan protocol and the telemedicine platform). Use of the protocol is currently limited to second or third trimester examinations as the sweeps are solely transabdominal, and first trimester examinations may require an additional transvaginal examination for optimal visualization. A video of the protocol, the training poster, and sample sweeps are available as supplemental material (Supplemental Materials 1, 2, 3, 4). In total, a VSI examination takes about 10–20 min to perform, less than 5 min to upload with standard internet, and 5–10 min to interpret.

### Telediagnostic system

The technical specifications of the “Medical for Ultrasound” (Med4US) telemedicine platform used in this study were previously published [26]. Briefly, this comprehensive telemedicine platform is installed on a tablet capable of sending imaging acquisitions over very low bandwidths, roughly equivalent to dial-up internet, with reasonable transmission times [26]. The software prompts the operator to input the patient’s clinical information, guides them in performing each of the 8 sweeps, then compresses, encrypts, and uploads the imaging data to the cloud. In the absence of an internet connection, images are saved locally and can later be uploaded. Remote interpretation by a specialist can subsequently be performed asynchronously. Clips are not viewed as video but rather as scrollable image stacks. The structured diagnostic report generated can then be sent back to the tablet and shared with the provider and patient.

### Testing the obstetric telediagnostic system

This study was conducted in accordance with the Declaration of Helsinki and approved by the Institutional Review Board (IRB) at the Hospital Nacional Docente Madre Nino San Bartolome on 2/6/2018.

Two operators, a nurse and a care technician, with no prior ultrasound experience, underwent an 8 h training on the protocol and use of the telemedicine software. Training involved approximately 4 h of didactic sessions, containing visual and video aids to teach the operators the external body landmarks of interest, the pattern of ultrasound sweeps across the abdomen, and the techniques for scanning. This was followed by approximately 4 h of hands-on training to practice and reinforce the protocol until the operators could comfortably and accurately reproduce it independently.

Second or third trimester patients over age 18 who presented to the Conde de la Vega Health Center in Lima, Peru were recruited from 6/2018–3/2019. All patients participated in the informed consent process and then underwent a SOC ultrasound exam performed by a radiologist followed by VSI ultrasound using telediagnostic system performed by one of the two previously ultrasound-naïve operators who had undergone training. The acquired VSI clips were uploaded to the cloud for remote interpretation at the time of their acquisition but were not immediately accessed and reviewed during the recruitment phase of the study, and no feedback was provided to the ultrasound-naïve operators.

All exams were performed using a portable Mindray DP-10 (Mindray, China), set to the Obstetrics preset, with a 4.5 mHz curvilinear transducer. For VSI exams, this was attached to a Windows 10 tablet containing the telemedicine software. A low- to moderate-speed, 3G internet connection in the health clinic allowed upload times within 5 min.

### Image interpretation

A single Peruvian radiologist acquired the SOC images, interpreted them, and generated a report per standard guidelines [27, 28]. The radiologist did not view the VSI scans at any point. VSI images were interpreted remotely by a Maternal-Fetal Medicine fellow in the United States who was blinded to the results of SOC ultrasound. VSI images were evaluated for ability to confirm a live fetus (based on cardiac activity), fetal number, fetal presentation, placental location, and amniotic fluid volume. Image quality was assigned using 3-point Likert scale (1-“excellent,” 2-“acceptable,” or 3-“poor”) as was reader confidence in imaging findings (1-“confident,” 2-“intermediate,” 3-“not confident”).

This version of the Obstetric VSI protocol was not designed for estimation of fetal biometry because the proper imaging planes needed for accurate and reproducible biometric measurements are not always present in the cine clips. However, as an exploratory outcome, we investigated three methods of estimating fetal gestational age using the protocol to identify gross discrepancies in fetal size requiring further follow up. These included 1) a gross visual estimation of fetal gestational age (second versus third trimester), 2) fetal biometry using strict criteria, and 3) fetal biometry using relaxed criteria. A strict measurement was an optimal biometric measurement in the correct plane closely or perfectly fulfilling current guidelines [29]. A relaxed measurement was a close estimate when the measurement of interest was not in the optimal plane or orientation.

After initial analysis, cases that were discrepant between VSI ultrasound and SOC ultrasound underwent adjudication by an experienced Maternal-Fetal Medicine physician to determine the most accurate interpretation.

### Statistical analysis

Agreement on categorical variables was assessed by overall agreement between VSI and SOC ultrasound and Cohen’s kappa. Resultant kappa values were compared to a theoretical mean of zero using a one-sample t-test to determine whether the kappa value was significantly better than zero. Biometry was compared between VSI and SOC ultrasound using paired t-tests, calculation of ICC, and Bland-Altman analysis. ICC values were calculated using a two-way mixed effects model for absolute agreement. ICC was classified as “excellent” with values greater than 0.9, “good” with values between 0.75 and 0.9, “moderate” with values between 0.5 and 0.75, and “poor” with values less than 0.5 [30]. Both ICC values and Bland-Altman bias were independently compared to a theoretical mean of zero using a one-sample t-test. These analyses were repeated separately by trimester and by the number of biometry measurements achievable on VSI. Sweep length was compared between cases with “acceptable” versus “excellent” image quality using unpaired t-tests. Differences in sweep length based on the number of biometry measurements were assessed with ordinary one-way ANOVA with Tukey’s test used for pairwise comparisons. All statistical analysis was performed using MATLAB (v2019b, The Mathworks, Inc., Natick, MA) and SPSS (v26, IBM Corporation, Armonk, NY).

## Results

**Fig. 3:**
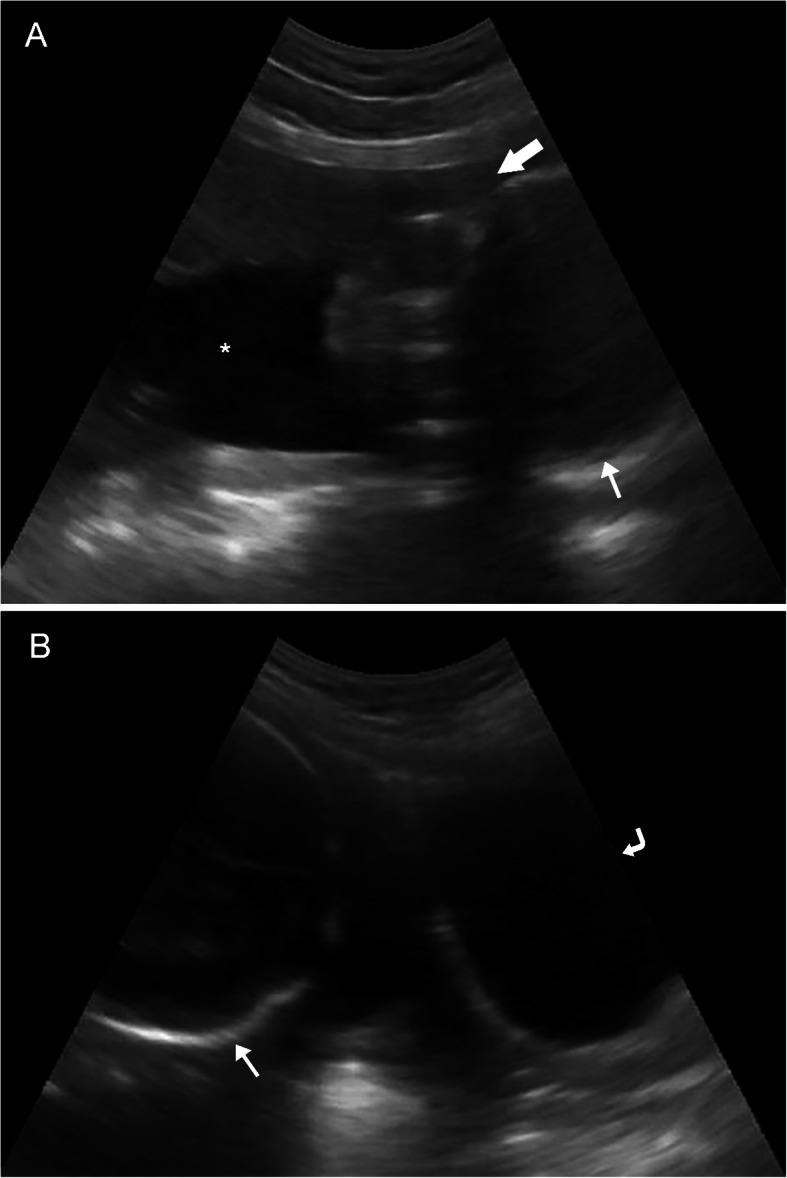
Example Images Acquired by Individuals Without Prior Training. **a**, **b** Still images from an ultrasound cine clip of a sagittal VSI sweep across the lower uterine corpus obtained by an operator without prior ultrasound training demonstrating a singleton early third trimester fetus in vertex presentation (thin arrow) with an anterior-mid placenta (block arrow) and grossly normal amniotic fluid volume (*). Maternal bladder is also seen in **b** (curved arrow)

**Fig. 4:**
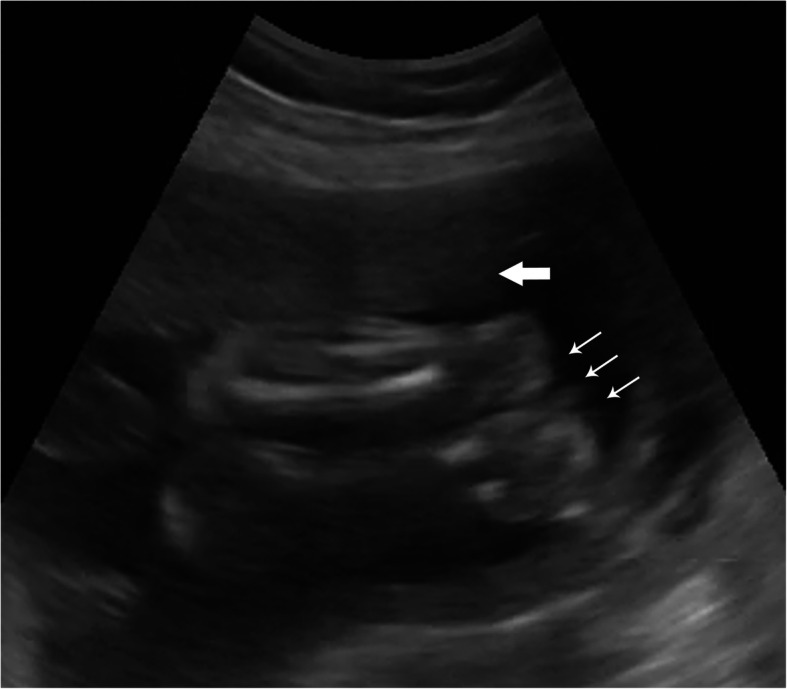
Example Non-cephalic Presentation. Still image from a VSI sweep of a singleton second trimester fetus in breech presentation (small arrows indicate fetal feet and lower extremities as presenting parts). The placenta is seen anterior excluding a low-lying placenta or placenta previa (block arrow)

One hundred twenty-six patients (inclusive of 68 second trimester and 58 third trimester pregnancies) with a mean age of 25.7 years participated in the study. All images were successfully uploaded using the health clinic’s low- to moderate-bandwidth internet connection and were accessed in the United States for remote interpretation. Only one VSI imaging study was rated of “poor” imaging quality (visualization of an early second trimester fetus was limited by maternal body habitus). The remaining imaging clips (> 99%) provided a diagnostic exam of “acceptable” (38.1%) or “excellent” (61.1%) image quality. The overwhelming majority of sweeps were performed correctly (suboptimal sweep position was only identified in two exams). Sample images and clips obtained from the VSI protocol and uploaded by the telediagnostic system are seen in Figs. 3, 4 and Supplemental Materials 3, 4. The average file size of uncompressed clips was 12.5 megabytes and average sweep time was 12.6 seconds. Sweeps 3 (*p* = 0.0006) and 7 (*p* = 0.01) of the protocol were significantly longer for cases with excellent image quality. No differences in sweep length based on the total number of attained biometric measurements were found.

Results for overall agreement between VSI and SOC ultrasound are shown in Table 1. There was excellent agreement for number of fetuses (100% agreement), fetal presentation (95.8% agreement, *κ* =0.78 (*p* < 0.0001)), placental location (85.6% agreement, *κ* =0.74 (*p* < 0.0001)), and assessment of amniotic fluid volume (99.2% agreement). When fundal placental position was counted as either anterior or posterior, the agreement improved to 94.4%. The reader was highly confident in these assessments (for all: median confidence rating “3” corresponding to “confident” on the 3-point Likert scale). VSI was able to confirm the presence of a live fetus in 76.2% of cases. In the cases without a confirmed live fetus, a full cardiac cycle was not able to be visualized secondary to fast sweep speed or early second trimester fetus.

**Table 1:** Agreement between volume sweep imaging protocol and standard of care ultrasound

Variable^a^	Volume sweep imaging^b^	Standard of care^b^	Overall agreement	Cohens kappa^c^	Confidence^d^
Live Fetus	76.2% (67.8–83.3%)	100% (97.1–100%)	76.2%	…^e^	3 (1–3)
Number of Fetuses (% single)	100% (97.1–100%)	100% (97.1–100%)	100%	…^e^	3 (2–3)
Fetal Presentation (% vertex)	87.3% (77.3–94%)	91.5% (82.5–96.8%)	95.8%	0.78 (0.53–1.00, *p* < 0.0001)	3 (1–3)
Placental position					
Anterior Placenta	52.0% (42.9–61.0%)	50.4% (41.3–59.5%)	85.6% (94.4%^f^)	0.74 (0.63–0.85, *p* < 0.0001)	^g^
Posterior Placenta	42.4% (33.6–51.6%)	40.0% (31.3–49.1%)			
Fundal Placenta	5.6% (2.3–11.2%)	9.6% (5.1–16.2%)			
Placenta Previa	4.0% (1.3–9.1%)	0% (0–2.9%)	96.0%	…^e^	3 (1–3)
Placenta Previa (consensus read)	3.2% (0.9–8.0%)		96.8%		
Amniotic Fluid Volume (% normal)	99.2% (95.7–100%)	100% (97.1–100%)	99.2%	…^e^	3 (1–3)
Normal Exam	92.1% (85.9–96.1%)	96.8% (92.1–99.1%)	95.2%	0.55 (0.20–0.90, *p* < 0.0001)	3 (1–3)
Normal Exam (consensus read)	92.9% (86.9–96.7%)		96.0%	0.60 (0.25–0.94, *p* < 0.0001)	
Follow-up Recommendation (% normal)	99.2% (95.7–100%)	100% (97.1–100%)	99.2%	…^e^	3 (1–3)

^a^for categories in which consensus reads resulted in changes, these are noted below the original variable

^b^values are percentage (95% confidence interval)

^c^values are kappa (95% confidence interval, *p* value)

^d^confidence is specified as median (range)

^e^not defined, due to Peruvian radiologist labelling all identically

^f^agreement when fundal location included as agreeing with either anterior or posterior location

^g^not given

The vast majority of examinations were interpreted as normal with high agreement between VSI and SOC ultrasound (95.2% agreement, *κ* =0.55 (*p* < 0.0001)). For abnormal cases, there was 100% agreement (4/4 cases) for non-cephalic presentation in the third trimester with high confidence by VSI reader for all assessments. VSI identified 5 cases (4 after adjudication) of possible low lying/placenta previa which were not reported on SOC ultrasound. All were second trimester fetuses with the potential for re-imaging later in pregnancy for confirmation. VSI identified 1 case of suspected oligohydramnios in a third trimester gestation (verified on adjudication) which was not reported on SOC ultrasound.

Gross visual estimation of fetal gestational age by trimester (with fetuses straddling the trimester definition (26–30 weeks) counted as agreement if reported as either second or third) yielded only 5 disagreements. Disagreements differed by only a few days outside of defined ranges for each trimester. After adjudication, all 5 discrepant cases were grouped into the correct trimester yielding 100% agreement.

**Fig. 5:**
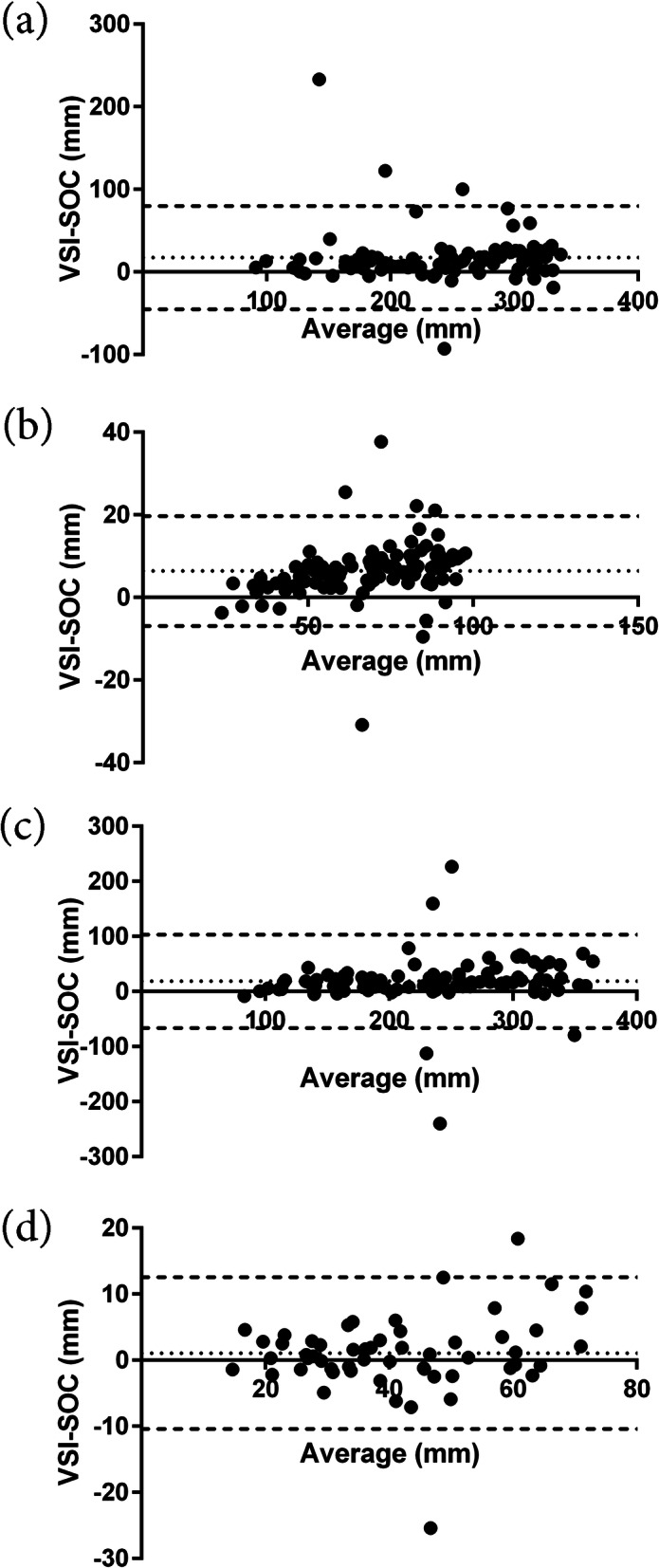
Bland-Altman plots of agreement in measurements of **a** head circumference; **b** biparietal diameter, **c** abdominal circumference; **d** femur length. Y-axis: mean difference in biometry measurement between volume sweep imaging (VSI) and standard of care (SOC) imaging and X-axis: mean biometry measurement of VSI and SOC. Dashed line representing 95% limits of agreement of measurements and dotted line representing Bland-Altman bias

ICC between VSI and SOC measurements was good-excellent for individual biometric measurements (ICC = 0.81–0.93) and excellent for overall estimated gestational age (EGA) (ICC = 0.95) when examining all exams (Table 2). For subgroups, second trimester demonstrated moderate correlation for individual measurements (ICC = 0.67–0.84) and excellent correlation for overall EGA (ICC = 0.94), and third trimester demonstrated poor - moderate correlation for individual measurements (ICC = 0.28–0.68) and moderate correlation for overall EGA (ICC = 0.64). VSI slightly over-estimated all biometric measurements except femur length and slightly overestimated EGA in all exams (Table 2). The magnitude of this difference is not likely to be clinically significant for the intended application of teleultrasound. Bland-Altman plots were constructed to identify any systematic differences between VSI and SOC ultrasound, and these demonstrated low-level fixed bias but also confirmed that the vast majority of measurements fell within 95% limits of agreement (Fig. 5). Bland-Altman bias was lowest for femur length (1.1 for all exams; 0.2 and 3.0 for second and third trimester subgroups, respectively) and highest for abdominal circumference (18.6 for all exams; 16.8 and 20.7 for second and third trimester subgroups, respectively) (Table 2). When examining overall EGA, Bland-Altman bias was lowest for second trimester calculations (6.5) and highest for third trimester calculations (15.2).

**Table 2:** Comparison of volume sweep imaging protocol biometry using strict criteria and standard of care ultrasound biometry

Variable	Volume sweep imaging^a^	Standard of care^a^	*p* value^b^	Intraclass correlation coefficient^c^	Bland-altman bias^c^
All Exams					
Biparietal Diameter (mm)	71.1 ± 20.3 (*n* = 111)	65.6 ± 18.7	< 0.0001	0.89 (0.50–0.96, *p* < 0.0001)	6.4 (−6.9–19.7, *p* < 0.0001)
Head Circumference (mm)	250.0 ± 66.3 (*n* = 99)	236.0 ± 68.0	< 0.0001	0.86 (0.71–0.92, *p* < 0.0001)	17.5 (−45.0–79.9, *p* < 0.0001)
Abdominal Circumference (mm)	243.0 ± 77.8 (*n* = 102)	224.0 ± 74.8	< 0.0001	0.81 (0.69–0.88, *p* < 0.0001)	18.6 (−66.2–103, *p* < 0.0001)
Femur Length (mm)	41.8 ± 16.5 (*n* = 56)	46.3 ± 15.8	0.17	0.93 (0.88–0.96, *p* < 0.0001)	1.1 (−10.4–12.6, *p* = 0.17)
Estimated Gestational Age (days)	195 ± 49 (*n* = 109)	186.0 ± 46.0	< 0.0001	0.95 (0.69–0.98, *p* < 0.0001)	10.4 (−10.6–31.5, *p* < 0.0001)
Second Trimester Exams					
Biparietal Diameter (mm)	56.2 ± 14.0	51.6 ± 13.0	< 0.0001	0.84 (0.54–0.93, *p* < 0.0001)	4.6 (−7.7–16.9, *p* < 0.0001)
Head Circumference (mm)	202.0 ± 45.3	189.0 ± 47.6	0.0007	0.84 (0.69–0.91, *p* < 0.0001)	11.4 (−35.5–58.4, *p* < 0.0001)
Abdominal Circumference (mm)	187.0 ± 50.8	168.0 ± 46.7	0.001	0.67 (0.45–0.80, *p* < 0.0001)	16.8 (−55.0–88.5, *p* < 0.0001)
Femur Length (mm)	32.7 ± 9.6	34.6 ± 10.8	0.87	0.83 (0.70–0.91, *p* < 0.0001)	0.2 (−10.8–11.1, *p* = 0.87)
Estimated Gestational Age (days)	158.0 ± 27.2	150.0 ± 27.0	< 0.0001	0.94 (0.65–0.98, *p* < 0.0001)	6.5 (−6.9–19.9, p *p* < 0.0001)
Third Trimester Exams					
Biparietal Diameter (mm)	90.0 ± 6.79	81.9 ± 7.7	< 0.0001	0.33 (−0.10–0.64, *p* < 0.0001)	8.7 (−4.6–22.0, *p* < 0.0001)
Head Circumference (mm)	313.0 ± 22.6	291.0 ± 42.7	0.0001	0.38 (0.06–0.62, *p* = 0.001)	25.3 (−50.8–101, *p* < 0.0001)
Abdominal Circumference (mm)	309.0 ± 44.7	289.0 ± 39.5	0.0007	0.28 (0.02–0.52, *p* = 0.015)	20.7 (−77.8–119, *p* < 0.0001)
Femur Length (mm)	61.4 ± 9.5	59.9 ± 7.7	0.051	0.68 (0.32–0.87, *p* < 0.0001)	3.0 (−8.97–15, *p* = 0.051)
Estimated Gestational Age (days)	242.0 ± 23.0	229.0 ± 18.4	< 0.0001	0.64 (−0.02–0.86, *p* < 0.0001)	15.2 (−9.46–39.9, *p* < 0.0001)

^a^values are mean ± standard deviation

^b^*p* value using paired t-test

^c^values are kappa (95% confidence interval, *p* value)

**Fig. 6:**
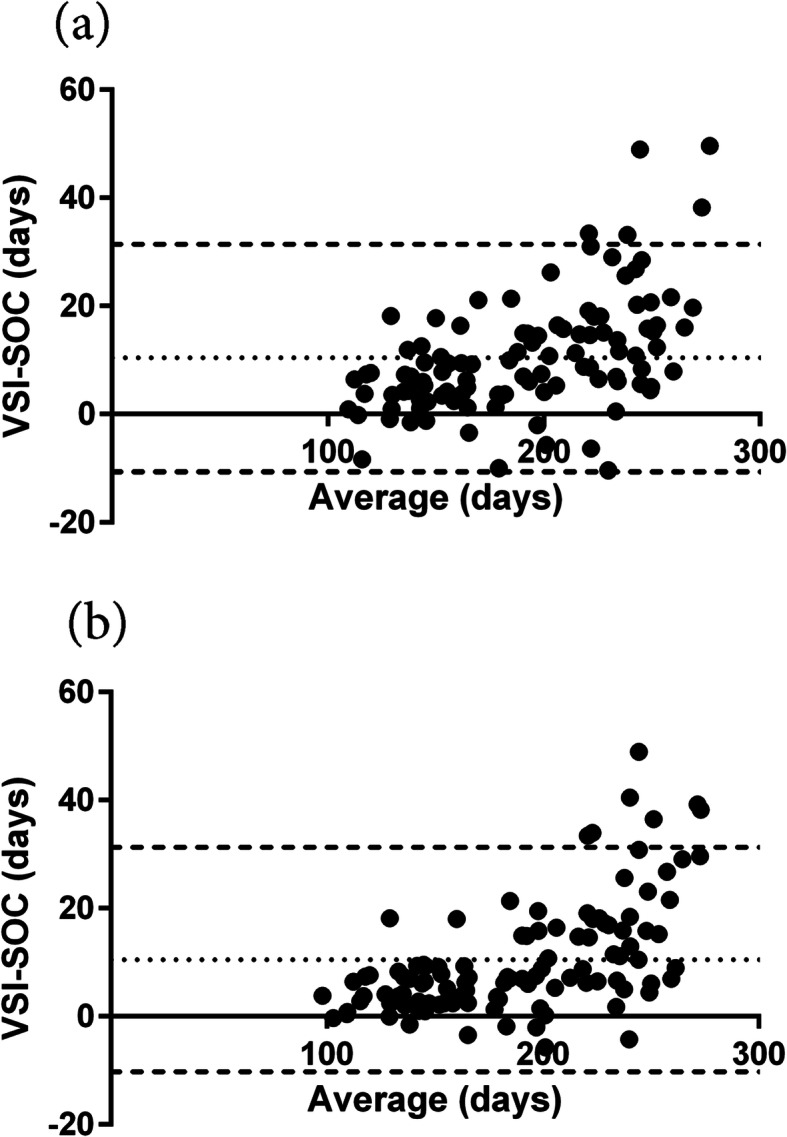
Bland-Altman plots of agreement in measurements of **a** strict biometry measurements and **b** relaxed (inclusive of strict) biometry measurements. Y-axis: mean difference in biometry measurement between volume sweep imaging (VSI) and standard of care (SOC) imaging and X-axis: mean biometry measurement of VSI and SOC. hashed line representing 95% limits of agreement of measurements and dotted line representing Bland-Altman bias

There were no significant differences in agreement of EGA noted between VSI and SOC ultrasound when using strict versus relaxed biometry measurements (Table 3, Fig. 6). Table 4 summarizes the differences in biometric measurements between VSI and SOC ultrasound. Among all exams and all biometry measurements, 69.1% of all the VSI examinations and 88.5% of second trimester exams showed agreement within 2 weeks to SOC ultrasound measurements. Achieving 4/4, 3/4, 2/4, or 1/4 of possible biometric measurements by VSI did not impact agreement with SOC ultrasound.

**Table 3:** A comparison of strict versus relaxed biometry measurements obtained using volume sweep imaging protocol

Variable	All measurements (*n* = 109)	Second trimester only (*n* = 68)	Third trimester only (*n* = 58)
Number of biometry measurements achieved (strict)	2 (0–4)	2 (0–4)	2 (0–4)
Number of biometry measurements achieved (including relaxed)	3 (0–4)	3 (0–4)	3 (0–4)
Estimated gestational age (days)			
Strict^a^	195 ± 49 (*n* = 109)	158 ± 27 (*n* = 60)	242 ± 23 (*n* = 49)
Including relaxed^a^	194 ± 51 (*n* = 122)	155 ± 28 (*n* = 67)	243 ± 24 (*n* = 55)
p-value^b^	0.37	0.44	0.58

^a^ values are mean ± standard deviation

^b^using paired t-test

**Table 4:** Comparison of strict versus relaxed biometry criteria and calculated estimated gestational age by volume sweep imaging protocol and standard of care ultrasound

Trimester	Within 7 days		Within 14 days	
	Overall agreement (strict)	Overall agreement (including relaxed)	Overall agreement (strict)	Overall agreement (including relaxed)
All exams (*n* = 126)	42.2% (32.8–52.0%)	48.2% (38.6–57.9%)	67.9% (58.3–76.5%)	69.1% (59.6–77.6%)
Second trimester (*n* = 68)	56.7% (43.2–69.4%)	63.9% (50.6–75.8%)	85% (73.4–92.9%)	88.5% (77.8–95.3%)
Third trimester (*n* = 58)	24.5% (13.3–38.9%)	28.6% (16.6–43.3%)	46.9% (32.5–61.7%)	44.9% (30.7–59.8%)

All values are percentage (95% confidence interval)

## Discussion

The Obstetric ultrasound telediagnostic system with asynchronous reads presented in this study removes substantial barriers to Obstetric ultrasound access in LMICs. For the price of a tablet and portable ultrasound, individuals with a few hours of structured hands-on training are capable of acquiring imaging acquisitions allowing the identification of key information about a pregnancy including confirmation of a live fetus, fetal number, fetal presentation, placental location, and amniotic fluid volume. Pregnancy complications which may be identified by this type of ultrasound, including multiple gestation, non-cephalic presentation in the third trimester, abnormal amniotic fluid volume, and low lying placenta/placenta previa, can be detected prior to labor and delivery, allowing transfer to a higher level of care and possible prevention of adverse outcomes. Barriers to deployment of standard ultrasound capability including cost, technical complexity, lack of specialists/sonographers, lack of infrastructure, and poor internet connections are all overcome with this approach. Thus, this telediagnostic Obstetric ultrasound system provides a scalable, effective, and potentially life-saving means to deliver Obstetric ultrasound to low resource areas.

Lowering the maternal mortality rate is a vital public heath goal as stated in Millennium Development Goal 5 and has critical downstream impacts on family health, social, and economic conditions [31]. Obstetric telediagnosis has the potential to contribute to achieving this goal through early identification of pregnancy complications that may lead to preventable maternal morbidity or mortality. While we have proven that this approach is feasible, elucidating where exactly this approach fits into healthcare delivery will require additional study using a dissemination and implementation framework [32]. Given the heterogeneity of systems and communities, using a framework helps account for the needs and considerations of individual communities in the design and implementation of telediagnostic Obstetric ultrasound. Research and development of a robust referral system to bring women with ultrasound-identified pregnancy complications from their rural setting to a hospital center will be crucial alongside this telemedicine system.

Both operators received no formal quality assessment or feedback over the course of the study after their initial training. Since the data collection spanned a year, the consistent quality of the scans shows retention of the protocol. Although imaging quality was already “acceptable” or “excellent” in greater than 99% of cases, regular feedback could assist in improving quality even further. In the scans rated as “acceptable” instead of “excellent,” involved factors included insufficient depth in the third trimester, suboptimal maternal body habitus, and small uterine size in the second trimester. Even in the lower image quality exams where biometry could not be performed or live fetus could not be confirmed, other more important parameters such as fetal number and placental location were able to be reliably assessed. Future adaptations may consider modifying the protocol for fetuses earlier than 20 weeks.

While disagreements between VSI and SOC ultrasound were rare, only a few reference ultrasound images were saved limiting our ability to delineate the source of disagreement. Additionally, the majority of exams included in this study contained normal findings, as would be expected based on known prevalence of the types of pregnancy complications that are detectable by ultrasound. Therefore, further investigation must be undertaken in a patient group with abnormal findings to ensure the protocol performs equally well in detection of pregnancy complications such as malpresentation, placenta previa, multiple gestation, and abnormal fluid volume. Additional areas for future investigation include explorations of artificial intelligence including automated detection of fetal heart rate, estimation of gestational age, and fetal presentation.

It should be acknowledged that the Obstetric VSI protocol was not designed for assessment of fetal biometry. Despite this, exploratory use of the protocol for biometry in this study demonstrated high correlation in EGA by VSI compared to SOC ultrasound and found that using strict criteria versus relaxed criteria for fetal biometric measurements did not improve estimation of gestational age. These are encouraging results for use of the telediagnosis for gross estimation of fetal size, though the authors acknowledge that these results do not support the use of the protocol for more precise diagnosis of fetal growth abnormalities given the discrepancies found in third trimester biometry measurements in this study. In these cases, patients should be referred to a higher level of care with a trained sonographer. In the future, the addition of calibrated 3D reconstruction will allow the generation of images in the precise planes needed for accurate measurement according to standard guidelines.

## Conclusion

Our work demonstrates that a VSI protocol with teleultrasound system has substantial promise to increase access to prenatal ultrasound imaging worldwide. In this study, VSI showed excellent agreement with the SOC ultrasound in assessing vital descriptive features of a pregnancy and allowed for close estimation of gestational age. Large-scale use of this technology has the potential to improve access to high-quality prenatal care and improve maternal and neonatal/perinatal mortality and morbidity from preventable causes.

## Supplementary Information

**Additional file 1.** Obstetric Training Video.

**Additional file 2.** Obstetric VSI Protocol Training Poster. Poster used in the original Obstetric training session.

**Additional file 3.** Obstetric VSI Sweep of a Second Trimester Fetus. An example of a transverse sweep from the maternal pubic symphysis to the maternal xyphoid process at the midline. The sweep shows a singleton second trimester fetus in the vertex presentation, an anterior placenta position, and fetal cardiac activity. The amniotic fluid volume is grossly normal.

**Additional file 4.** Obstetric VSI Sweep of a Third Trimester Fetus. An example of a sagittal sweep from the maternal right anterior superior iliac spine to the maternal left anterior superior iliac spine with the inferior aspect of the ultrasound transducer at the maternal pubic symphysis. The sweep shows a singleton third-trimester fetus in breech presentation. Placenta is not visualized in the lower uterine segment or near the internal cervical os. The amniotic fluid volume is grossly normal.

## Data Availability

The dataset supporting the conclusions of this article is available in the Mendeley Data repository, (https://data.mendeley.com/datasets/4k443mtkxd/2). Toscano, Marika; Marini, Thomas; Drennan, Kathryn; Baran, Timothy; Kan, Jonah; Garra, Brian; Dozier, Ann; Ortega, Rafael; Quinn, Rosemary; Zhao, Tina; Egoavil, Miguel; Tamayo, Lorena; Carlotto, Claudia; Castaneda, Benjamin (2021), “Testing Telediagnostic Obstetric Ultrasound in Peru: A New Horizon in Expanding Access to Prenatal Ultrasound” , Mendeley Data, V2, doi: 10.17632/4k443mtkxd.2

## Abbreviations

VSI: Volume sweep imaging
SOC: Standard of care
LMICs: Low- and middle-income countries
ICC: Intraclass correlation coefficient
EGA: Estimated gestational age
