# Supplementary material for: Testing telediagnostic obstetric ultrasound in Peru: a new horizon in expanding access to prenatal ultrasound
Source: BMC Pregnancy Childbirth. 2021 Apr 26;21:328. doi: 10.1186/s12884-021-03720-w (PMC8074497; doi:10.1186/s12884-021-03720-w)
Supplement: Supplementary file 1 — Additional file 1. Obstetric Training Video. [file 12884_2021_3720_MOESM1_ESM.docx]

Supplemental Material 1. Obstetric Training Video.

Video is available for viewing or download from the following link: https://rochester.box.com/s/n526qrpftu394qby68o9ukd75e3p7pme
