# Supplementary figures and images for: Testing telediagnostic obstetric ultrasound in Peru: a new horizon in expanding access to prenatal ultrasound
Source: BMC Pregnancy Childbirth. 2021 Apr 26;21:328. doi: 10.1186/s12884-021-03720-w (PMC8074497; doi:10.1186/s12884-021-03720-w)

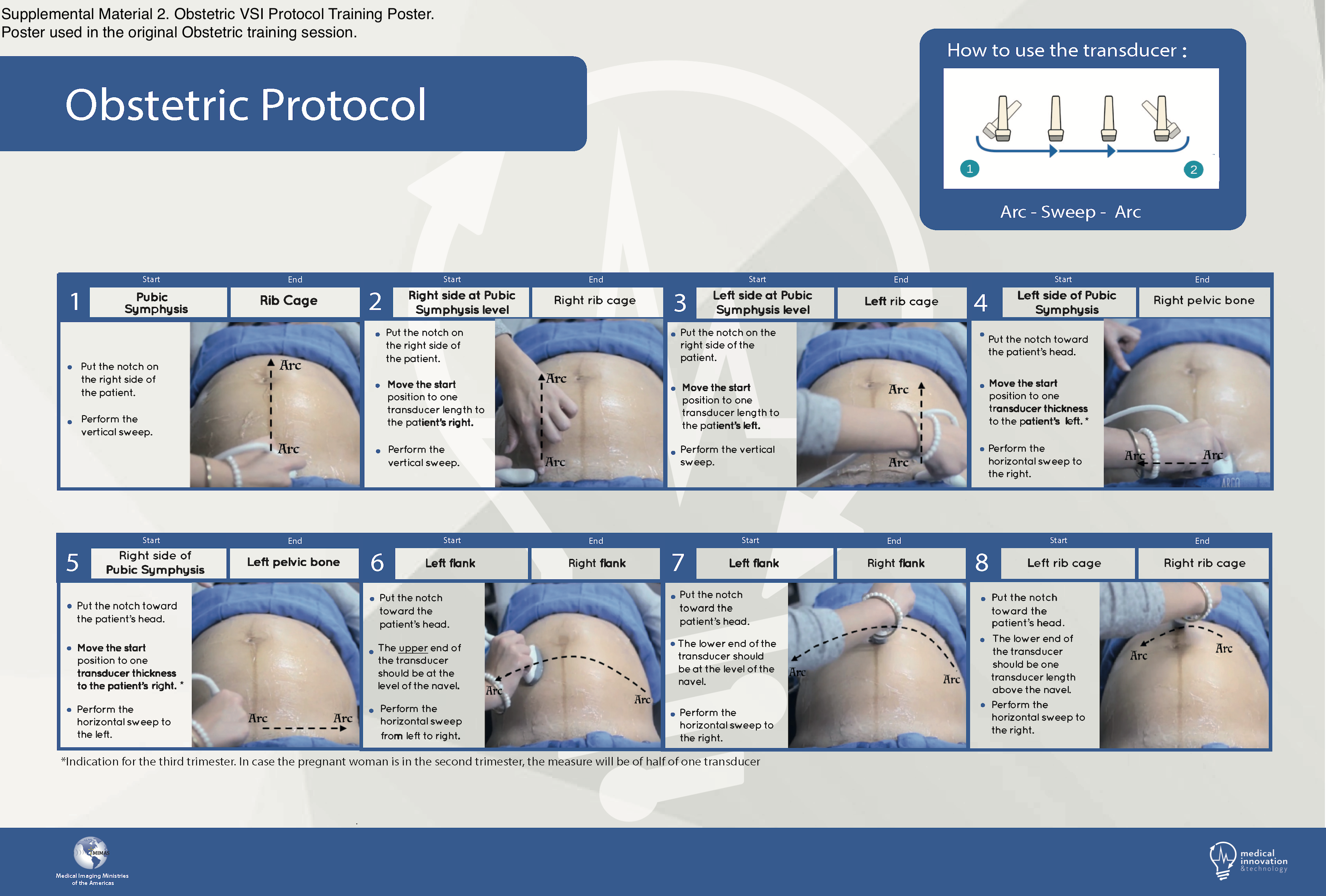

Supplement: Supplementary file 2 — Additional file 2. Obstetric VSI Protocol Training Poster. Poster used in the original Obstetric training session. [file 12884_2021_3720_MOESM2_ESM.tiff]
